# Individual and community-level factors associated with iron-rich food consumption among children aged 6–23 months in Rwanda: A multilevel analysis of Rwanda Demographic and Health Survey

**DOI:** 10.1371/journal.pone.0280466

**Published:** 2023-01-19

**Authors:** Habitu Birhan Eshetu, Mengistie Diress, Daniel Gashaneh Belay, Mohammed Abdu Seid, Dagmawi Chilot, Deresse Sinamaw, Wudneh Simegn, Abiyu Abadi Tareke, Abdulwase Mohammed Seid, Amare Agmas Andualem, Desalegn Anmut Bitew, Yibeltal Yismaw Gela, Anteneh Ayelign Kibret

**Affiliations:** 1 Department of Health Promotion and Health Behavior, Institute of Public Health, University of Gondar, Gondar, Ethiopia; 2 Department of Human Physiology, School of Medicine, College of Medicine and Health Sciences, University of Gondar, Gondar, Ethiopia; 3 Department of Human Anatomy, School of Medicine, College of Medicine and Health Sciences, University of Gondar, Gondar, Ethiopia; 4 Department of Epidemiology and Biostatistics, Institute of Public Health, University of Gondar, Gondar, Ethiopia; 5 Unit of Human Physiology, Department of Biomedical Science, College of Health Sciences, Debre Tabor University, Debre Tabor, Ethiopia; 6 College of Health Sciences, Center for Innovative Drug Development and Therapeutic Trials for Africa (CDT-Africa), Addis Ababa University, Addis Ababa, Ethiopia; 7 Unit of Human Physiology, Department of Biomedical Science, College of Health Sciences, Debre Markos University, Debre Markos, Ethiopia; 8 Department of Social and Administrative Pharmacy, School of Pharmacy, College of Medicine and Health Sciences, University of Gondar, Gondar, Ethiopia; 9 Amref Health Africa in Ethiopia, SLL Project, COVID-19 Vaccine /EPI Technical Assistant at West Gondar, Addis Ababa, Ethiopia; 10 Department of Clinical Pharmacy, School of Pharmacy, College of Medicine and Health Sciences, University of Gondar, Gondar, Ethiopia; 11 Department of Anesthesia, Wollo University, Dessie, Ethiopia; 12 Department of Reproductive Health, College of Medicine and Health Sciences, University of Gondar, Gondar, Ethiopia; Madda Walabu University, ETHIOPIA

## Abstract

**Background:**

Iron-rich food consumption has an invaluable effect for neonatal and fetal brain development as well as metabolic activities. Despite the public health importance of the consumption of iron-rich foods, there was no study, that assessed iron-rich food consumption in Rwanda. Therefore this study aimed to assess iron-rich food consumption and associated factors among children aged 6–23 months using Rwanda Demographic and Health Survey (RDHS).

**Methods:**

Secondary data analysis was done using RDHS-2019/20. Total weighted samples of 2455 children aged 6–23 months were included. Data coding, cleaning, and analysis were performed using Stata 16. Multilevel binary logistic regression were performed to identify factors associated with iron-rich food consumption. Adjusted Odds Ratio (AOR) with a 95% CI, and p-value <0.05 were used to declare statistical significance.

**Results:**

The prevalence of good iron-rich food consumption was 23.56%(95% CI: 21.92,25.28). Northern province of Rwanda (AOR  =  0.26,95%CI: 0.15,0.46), mothers secondary education and above (AOR: 2.37, 95% CI: 1.41, 4.01), married mothers (AOR:1.31, 95% CI: 1.01,1.71), rich wealth status (AOR = 2.06, 95% CI: 1.48, 2.86), having post-natal visit (AOR = 1.45, 95% CI: 1.10,1.91), mothers media exposure (AOR: 1.75, 95% CI: 1.22, 2.52) and drugs given for intestinal parasite (AOR = 1.37, 95% CI: 1.04, 1.80) were associated with iron-rich food consumption.

**Conclusions:**

This study shows that overall iron-rich foods consumption was low in Rwanda. The residing in the North province, mother’s secondary and higher educational status, married marital status, rich and middle wealth status, having media exposure, drugs given for intestinal parasites, and having child’s post-natal checkup were variables significantly associated with iron-rich food consumption. The region-based intervention will improve the consumption of iron-rich food. In addition, health policies and programs should target educating mothers/caregivers, encouraging parents to live together, improving their wealth status, working on mass media access by the women, and encouraging mothers post-natal checkups to improve iron-rich food consumption.

## Background

Iron deficiency is the most common micronutrient deficiency, hitting more than two billion people worldwide, with African children bearing the most burden [1, 2]. Iron deficiency anemia (IDA) remains a major public health issue, particularly for children and women in low- and middle-income countries [3]. The World Health Organization (WHO) predicts that 800 million women and young children worldwide were anemic in 2011, and that increasing iron consumption may remove 42% of anemia in children and 50% of anemia in women [1, 4]. According to a national survey, the prevalence of anemia among children aged 6–59 months in Rwanda shows that 37% [5], and the prevalence of iron deficiency anemia ranged from 3% to 88% among young children [6–9].

Iron is an important element in brain metabolism [10–12]. Iron deficiency can alter neurotransmitter balance, decrease myelin synthesis, impede synaptogenesis, and affect basal ganglia function [10, 13–15]. In addition to cognitive functions and psychomotor development, IDA can lead to acute life-threatening conditions like tachypnea, palpitation, dyspnea, hypotension, and congestive heart failure, which leads to immediate hospitalization [16]. Evidence also shows that iron deficiency is a common comorbidity in autism spectrum disorder and attention deficit/hyperactivity disorder [17]. These long-term impacts can have a negative impact on learning ability and professional skill acquisition.

Children under the age of seven are the population group most sensitive to iron deficiency, especially children under the age of two are vulnerable to iron deficiency because of their rapid growth [3, 14]. As a result, limiting the advancement of iron deficiency is especially crucial during infancy and early childhood, when there is rapid growth and development, particularly of the brain [18], that increases the vulnerability to IDA-induced impairment.

Low consumption of iron-containing foods and consumption of foods that interfere with iron absorption, such as phytates, also increase the risk of iron deficiency [19]. Iron-rich food consumption improves the hemoglobin levels of the blood [20]. Evidence shows that the prevalence of iron-rich food consumption in young children ranges from 21.4% in developing countries to 90% in developed countries [21–24]. Some of the factors associated with good iron-rich food consumption were wealth status, maternal and paternal educational status, Antenatal and post-natal visits, and media exposures [23, 25–27].

Currently, IDA control methods include the use of iron treatment on a continuous or intermittent basis, the consumption of iron sprinkles or fortified foods and beverages, better food safety, and dietary diversity monitoring. There was no previous research on the consumption of iron-rich foods in Rwanda. Therefore, this study aimed to assess the prevalences of iron-rich food consumption and associated factors in Rwanda using the recent Rwanda Demographic and Health Survey (RDHS-2019/20), which has a paramount effect to develop intervention strategies based on the findings of the study to improve iron-rich food consumption across the country. This study may also assist policymakers, NGOs, global organizations, and researchers in identifying the factors in Rwanda that influence iron-rich food consumption to provide urgent interventional measures and resource allocation to enhance their behavior.

## Methods

### Study settings and data source

In this study, the analysis was conducted based on large representative secondary data from Rwanda Demographic and Health Survey (RDHS) -2019/20, which was collected between November 9, 2019, and July 20, 2020. Rwanda is a landlocked country in Eastern Africa, bordered by Uganda, Burundi, Tanzania, and the Democratic Republic of the Congo, and is administratively subdivided into Kigali City and four provinces (Eastern, Northern, Southern, and Western) [28]. The 2019/20 RDHS used a two-stage sample design with the initial step was to categorize sample sites (clusters) made up of enumeration areas (EAs). A total of 500 sites were chosen, (388 in rural areas and 112 in cities [29]. The second stage entailed systematic sampling of households in all of the designated EAs, a total of 13,000 households were included. All women aged 15 to 49 who were either permanent residents or visitors who live in the selected residences the night before the survey were eligible for the interview. For this study we used kids recorded data set file (KR file), and extract the dependent and the independent variables. A total weighted samples of 2455 children were included in the analysis.

### Study variables

#### Outcome variable

The dependent variable of this study was the consumption of iron-rich foods by children aged 6–23 months, which was categorized as good and poor consumption. According to the DHS guideline, the number of youngest living children 6–23 months living with their mother who consumed at least one food rich in iron at any time in 24 hours preceding the interview among four food items, egg, organ meat(liver, heart, or other organs), meat (beef, pork, lamb, chicken), and fish or shellfish were considered as good consumption, otherwise poor consumption [30].

#### Independent variables

The individual-level variables are sex of the child, age of mother and child, level of education of mother and father, number of ANC visits, place of delivery, child postnatal check within 2 months, taking of drugs for intestinal parasites, and wealth status, whereas, the community-level variables include community-level poverty, media exposure, distance from the health facility, region, and residence which was driven from individual-level variables. After combining and recoding the respondents’ exposure to newspaper/magazine, radio, and television, community-level media exposure was developed. Because the data were not normally distributed, the median was used, and the results were classified as low if less than 50% of respondents were exposed to at least one medium, and high if more than 50% of respondents were exposed to at least one medium.

### Data management and statistical analysis

In this study, Stata version 16 software was used for data analysis. Prior to analysis, the data were weighted to verify the DHS sample’s representativeness and to get trustworthy estimates and standard errors. Hence, the DHS data is hierarchical we used women’s individual sample weight (V005/1000000) throughout the analysis. For the descriptive results, we used cross-tabulations and summary statistics.

DHS datasets contain hierarchical data structures with individuals nested under geographical clusters (primary sampling units) where children aged 6 to 23 months were nested within a cluster. This may violate the assumptions of standard logistic regression models such as the equal variance and independence assumptions. Thus, four models were fitted: the null model (no explanatory variables), model I (individual-level factors), model II (community-level factors), and model III (combined individual and community-level components). Besides, the Intra-class Correlation Coefficient (ICC), Median Odds Ratio (MOR), and Likelihood Ratio test (LLR) values, as well as the deviation (-2LLR), were utilized for model comparison and fitness, respectively. Model III was chosen as the best-fitting model because of its lowest deviation.

Random effects or measures of variation of the outcome variables were estimated by the median odds ratio (MOR), Intra Class Correlation Coefficient (ICC), and Proportional Change in Variance (PCV). Taking clusters as a random variable, the MOR is defined as the median value of the odds ratio between the area at the highest risk and the clusters at the lowest risk clusters when randomly picking out two clusters. ${{MOR=e}^{0.95}}^{\sqrt{VA}}$ While, the ICC tells the variation of iron-rich food consumption between clusters, and is calculated as; $ICC=\frac{VA}{VA+3.29}*100\%$. Furthermore, the PCV shows the variation in the prevalence of iron-rich food consumption among children 6–23 months explained by factors and calculated as; $PCV=\frac{Vnull-VA}{Vnull}*100\%$ where; Vnull = variance of the empty model, and VA = area/cluster level variance [31, 32].

The fixed effects or measure of association were used to estimate the association between the likelihood of prevalence of iron-rich food consumption and individual and community levels variables Finally, in the multivariable analysis, adjusted odds ratios with 95%confidence intervals and a p-value of less than 0.05 were utilized to identify associated factors of iron-rich food consumption.


$$Log\left(\frac{\pi ij}{1-\pi ij}\right)=\beta o+\beta 1xij+\beta 2xij+\cdots uj+eij$$

Where, *πij*: the probability of iron-rich food consumption, 1−*πij*: the probability of iron-rich food consumption. ß0 is the intercept that is the effect on iron-rich food consumption when the effect of all predictor variables is absent. *β*1*xij* are individual and community level variables for the i^th^ individual in group j, respectively. The ß’s are fixed coefficients indicating a unit increase in X can cause a ß unit increase in the likelihood of iron-rich food consumption. The uj shows the random effect (effect of clusters on the mother’s choice to give iron-rich food) for the j^th^ clusters [31, 32].

## Ethical consideration

The ethical approval and permission to access the data were obtained from the DHS website www.measuredhs.com. All the ethical standards are available at https://goo.gl/ny8T6X.

## Results

### Sociodemographic characteristics of the participants

In this study, a total weighted sample of 2455 children aged 6–23 months were included. The median age of children was 14 months (IQR = 10–19 months). The majority of the children(83.59%) were from rural areas, and the majority of them were from the east province(26.78). More than half of the children(50.55%) were males. The majority of the children (43.70%) were from poor households family (Table 1).

**Table 1:** Socio-demographic and other variables of children with their respective caregivers in Rwanda (n = 2455).

Variables	Category	Weighted Frequency	Weighted percent
Child age in months	6–11	834	33.97
	12–17	822	33.50
	18–23	799	32.54
Child sex	Male	1241	50.55
	Female	1214	49.45
Residence	Urban	403	16.41
	Rural	2052	83.59
Mother’s age in years	*<* 20	127	5.16
	20–34	1590	64.77
	35–49	738	30.07
Region	Kigali city	332	13.52
	South province	530	21.60
	west province	572	23.31
	North province	363	14.80
	east province	657	26.78
Mother’s educational level	No education	243	9.91
	Primary	1554	63.32
	Secondary and above	657	26.77
Mother’s occupation	Not working	645	26.29
	Working	1810	73.71
Mother marital status	Married	1259	51.27
	Not married	1196	48.73
Father educational level	No education	679	27.67
	Primary	1316	53.59
	Secondary	460	18.74
Father occupation	Not working	535	21.81
	Working	1919	78.19
Wealth index	Poor	1073	43.70
	Middle	469	19.11
	Rich	913	37.18
Family size	<5	962	39.18
	5+	1493	60.82
Media exposure	Yes	1958	79.77
	No	497	20.23
Distance of health facility (getting medical help for self)	Big problem	585	23.82
	Not a big problem	1870	76.18
Number of ANC	0	148	6.03
	1–3	1182	48.15
	4+	1125	45.83
Birth order	1	642	26.16
	2–4	1288	52.46
	5+	525	21.38
Child is twin	Yes	87	3.52
	No	2368	96.48
Current breast feeding	Yes	2280	92.86
	No	175	7.14
Place of delivery	Home	159	6.50
	Health facility	2295	93.50
Child postnatal check within 2 months	Yes	449	18.27
	No	2006	81.73
Had diarrhea recently	Yes	598	24.35
	No	1857	75.65
Fever in the last two weeks	Yes	642	26.15
	No	1813	73.85
Short rapid breath	Yes	292	11.89
	No	2163	88.11
Drugs for intestinal parasites in last 6 months	Yes	1540	62.73
	No	915	37.27

### Random effect analysis

Due to the hierarchical nature of the DHS data, we assessed the clustering effect. The ICC in the null model was high in the random-effects analysis. This means that the variance between clusters accounted for around 19.89% of the variability in good iron-rich food consumption, whereas the remaining 80.11% was due to individual variation. The empty model’s higher MOR value suggested a significant difference in iron-rich food consumption between clusters. In the empty model, the MOR value was 2.36, showing that if we witnessed two children from two different clusters, a child in the cluster with high iron-rich food consumption was a 2.36 times higher likelihood of having good iron-rich food consumption as compared to a child within the cluster with lower iron-rich food consumption. Model fitness was also assessed using deviance, with the model with the lowest deviance chosen as the best-fit model, in this case, Model III with a deviance of. 2336.953 (Table 2).

**Table 2:** Random effect analysis and model comparison results.

Parameters	Null model	Model I	Model II	Model III
Community-level variance	0.82(.55,1.20)	.51 (.31,.84)	0.47(0.29,0.78)	0.42(0.23,0.71)
ICC	19.89%	13.42%	12.68%	11.41%
MOR	2.36	1.97	1.92	1.85
PCV	Ref	37.80%	42.68%	48.78%
Log likelihood	-1301.9334	-1186.5764	-1249.7927	-1168.4765
Deviance(-2LL)	2603.8668	2373.1528	2499.5854	2336.953

ICC: Intra Class Correlation Coefficient, MOR: Median Odds Ratio, PCV: Proportional Change in Variance.

### Iron-rich foods consumption and associated factors

The prevalence of iron-rich food consumption among children aged 6–23 months in Rwanda was 23.56% (95% CI: 21.92,25.28). Fish or shellfish was commonly consumed food (15.46%), whereas the liver, heart, and other organs were the least (1.22%) consumed iron-rich foods (Table 3).

**Table 3:** Iron-rich foods consumption among children aged 6–23 months in Rwanda (n = 2455).

Variables	Category	Weighted Frequency	Percent(95%CI)
Iron-rich food consumption in the last 24 hours	Good	578.4482364	23.56 (21.92, 25.28)
	Poor	1,876.4946	76.44 (74.72, 78.08)
Gave child egg in the last 24 hours	Yes	189.823378	7.73
	No	2,265.1195	92.27
Gave child meat (beef, pork, lamb, chicken, etc.) in the last 24 hours	Yes	99.1025755	4.04
	No	2,355.8403	95.96
Gave child liver, heart, other organs in the last 24 hours	Yes	30.0592362	1.22
	No	2,424.8836	98.78
Gave child fish or shellfish in the last 24 hours	Yes	379.523113	15.46
	No	2,075.4197	84.54

From the final model, residing in the North province was the community-level variable positively associated with iron-rich food consumption. Similarly, mother’s educational status, marital status, wealth status, media exposure, drugs given for intestinal parasites, and child post-natal checkup were the individual-level variables positively associated with iron-rich food consumption.

Accordingly, children who live in the North province were 74% less likely to consume iron-rich food (AOR  =  0.26(CI: 0.15,0.46) compared to those who live in Kigali city. Children whose mothers had secondary education and above had (AOR: 2.37, 95% CI: 1.41, 4.01) higher odds of iron-rich food consumption compared to no education. Children whose mother is married had higher odds of iron-rich food consumption compared with unmarried ones (AOR:1.31, 95% CI: 1.01,1.71)). Children from rich families had (AOR = 2.06, 95% CI: 1.48, 2.86) higher odds of iron-rich food consumption compared with poor household families. Children whose mothers attended media had (AOR: 1.75, 95% CI: 1.22, 2.52) higher odds of iron-rich food consumption compared to their counterparts. Children who received drugs for intestinal parasites had (AOR = 1.37, 95% CI: 1.04, 1.80) higher odds of iron-rich food consumption compared to their counterparts. Children who had post-natal checkups had (AOR = 1.45, 95% CI: 1.10,1.91) higher odds of iron-rich food consumption compared to no post-natal checkups (Table 4).

**Table 4:** Multilevel regression analyses of good iron-rich food consumption in Rwanda (n = 2455).

Variables		Model I AOR (95% CI)	Model II AOR (95% CI)	Model III AOR (95% CI)
Child age in months	6–11	1		1
	12–17	0.82(0.60,1.08)		0.85(0.63,1.14)
	18–23	1.02(0.74,1.40)		1.04(0.76, 1.43)
Child sex	Male	1.17(0.94,1.45)		1.19(0.96,1.47)
	Female	1		1
Residence	Urban		1.52(1.04,2.21)	0.99(0.67,1.47)
	Rural		1	1
Region	Kigali city		1	1
	South		0.87(0.55,1.38)	0.84(0.53,1.35)
	West		0.69(0.44, 1.09)	0.70(0.44,1.11)
	North		0.29(0.16,0.50)	0.26(0.15,0.46)***
	East		0.72(0.46,1.12)	0.69(0.44,1.09)
Mother’s age in years	*<* 20	1		1
	20–34	0.99(0.60,1.65)		0.95(0.57,1.58)
	35–49	1.21(0.69,2.14)		1.12(0.63,1.98)
Mother’s educational level	No education	1		1
	Primary	1.47(0.92,2.35)		1.49(0.93,2.39)
	Secondary and above	2.34(1.39,3.95)		2.37(1.41, 4.01)**
Mother’s Occupation	Not working	1		1
	Working	0.94(0.72,1.23)		1.02(0.78,1.34)
Mother marital status	Unmarried	1		1
	Married	1.25(0.96,1.62)		1.31(1.01,1.71)*
Father Educational level	No education	1		1
	Primary	0.80(0.57,1.12)		0.84(0.60,1.18)
	Secondary and above	1.37(0.90, 2.09)		1.44(0.95,2.17)
Father Occupation	Not working	1		1
	Working	1.11(0.76, 1.60)		1.11(0.77,1.60)
Wealth Index	Poor	1	1	1
	Middle	1.32(0.95,1.81)		1.22(0.87,1.71)
	Rich	2.46(1.83, 3.29)		2.06(1.48, 2.86)***
Family Size	5	1		1
	5+	0.71(0.56,0.91)		0.71(0.62, 1.92)
Media Exposure	Yes	1.92(1.35, 2.71)		1.75(1.22, 2.52)**
	No	1		1
Distance of health facility (getting medical help for self)	Big problem		1	1
	Not a big problem		1.22(0.93, 1.60)	0.99(0.75,1.31)
Number of ANC	No at all	1		1
	1–3	1.22(0.72, 2.04)		1.31(0.78,2.20)
	4+	1.33(0.78, 2.41)		1.44(0.82, 2.44)
Place of delivery	Home	1		
	Health facility	0.93(0.55, 1.56)		0.91(0.54,1.52)
Community poverty	High		0.61(0.46, 0.82)	0.89(0.66,1.21)
	Low		1	1
Community media exposure	High		1.55(1.17,2.05)	1.17(0.87,1.58)
	low		1	1
Current Breast Feeding	Yes	0.87(0.59, 1.37)		0.92(0.61, 1.37)
	No	1		1
Drugs for intestinal parasites in last 6 months	Yes	1.29(0.98, 1.69)		1.37(1.04,1.80)*
	No	1		1
Short rapid breath	Yes	1.21(0.85, 1.63)		1.22(0.87, 1.66)
	No	1		1
Child postnatal check within 2 months	Yes	1.62(1.24, 2.13)		1.45(1.10,1.91)**
	No	1		1

NB

* = Significant at P-value 0.05

** = Significant at P-value 0.01

*** = Significant at P-value 0.001, CI Confidence Interval, AOR Adjusted Odds Ratio

## Discussion

Iron is a necessary micronutrient for a child’s growth and development. Due to an increased need for iron during periods of rapid growth, the World Health Organization recommends iron supplementation in young children [33]. This study aimed to assess the individual and community-level factors associated with iron-rich food consumption among children aged 6–23 months in Rwanda. In this study, the prevalence of iron-rich food consumption was 23.56%. The result is similar to a study conducted in Ethiopia 24.41% [21]. However, the finding of this study is lower than a study conducted in Mexico (63.1%) [24], East Asia and the Pacific (62.5%) [34], China (51%) [35], Bangladesh (48%) [36], Australia (82.6%) [37], and Ireland (90%) [22]. The lower prevalence of iron-rich food consumption in Rwanda and the above study might be due to the difference in the literacy level [38], which may influence their knowledge and in return the consumption of iron-rich food in Rwanda and the other countries. The other difference might be due to sociocultural and socioeconomic status, the above high prevalence of iron-rich food consumption might be due to the production of iron-rich food sources, for instance, those countries were among the high producers of iron-rich foods like meat [39].

In this study, residing in the North province was the community-level variable positively associated with iron-rich food consumption. Similarly, mother’s educational status, marital status, wealth status, media exposure, drugs given for intestinal parasites, and child post-natal checkup were the individual-level variables positively associated with iron-rich food consumption.

This study revealed that the odds of iron-rich food consumption among children who live in the North province were lower compared to children who live in Kigali city. This might be due to the difference in lifestyle, sociocultural, and awareness of iron-rich foods, hence those who live in cities are more likely to have better access to media and other advanced websites, which ultimately increases their ability to read and comprehend the nutritional guidelines [40]. This implies that there is a need for region-based interventions to improve iron-rich food consumption.

This study showed that the odds of iron-rich food consumption among children whose mothers were married were higher compared with unmarried mothers. Possible explanations could be that married mothers might have better coordination with their spouse, an increase in home effort, and receive better information from their spouse. This shows that encouraging parents to live together will improve the consumption of iron-rich food.

This study revealed that the odds of iron-rich food consumption among children from rich families were higher compared with poor families. The possible explanation could be children from poor households have poor access to adequate food, which makes them unable to get diverse food sources for the consumption of iron-rich food [41]. It indicates that resource determines the ease with which resources may be accessed to meet one’s own needs and, thus, children’s iron-rich food consumption might be compromised due to limited resources in the household. This suggests that there is a need to boost household wealth.

The odds of iron-rich food consumption among children with media-exposed families were higher compared to no media exposure. This is consistent with a study done in sub-Sahara Africa [23], and a systematic review [42]. Media has a role in expanding caregivers’ access to health messages on optimal feeding practices for their children and has been demonstrated to be extremely useful in enhancing mothers’ knowledge and behaviors on infant and young child feeding [43, 44]. This implies that working on media access by women will improve iron-rich food consumption in the country.

In this study, the odds of iron-rich food consumption among children who had a post-natal checkup within 2 months were higher compared to their counterparts. This is in line with the previous study [23, 25, 26, 45]. The possible explanation might be because during a postnatal check within 2 months, moms will have the opportunity to learn about healthy child nutrition and suitable feeding practices, as well as be motivated to nourish their children with iron-rich foods, hence they are more likely to accept suggestions by health professionals [23, 26]. This implies that there is a need to encourage mothers to attend post-natal visits when they gave birth as much as possible.

The odds of iron-rich consumption among children who received drugs for intestinal parasites in the last 6 months were higher compared to those who did not receive drugs. This finding is in agreement with a study done in sab-Sahara Africa [23]. This could be because women do have an opportunity to contact healthcare professionals and so receive counseling on healthy child nutrition, the impacts of the parasite on anemia, and how to treat parasite-related anemia during their visit to health institutions, thus mothers might have a strong desire and commitment to providing iron-rich foods for their children.

Regarding the strengths, the study uses nationally representative data in Rwanda countries, which is representative across the countries. This study also used a multilevel modeling technique to provide a more credible result that takes into consideration the hierarchical nature of the survey data. However, the study is not free of drawbacks. The survey is susceptible to social desirability due to the self-reported nature of the interview, and the study’s cross-sectional design may not explain the temporal association of the independent and outcome variables. The other limitation of this study is it only accounts animal sources of iron-rich food, which may affect the prevalence of iron-rich food consumption.

## Conclusions

This study shows that overall iron-rich foods consumption was low in Rwanda. Residing in the North province, mother’s secondary and higher educational status, married marital status, rich and middle wealth status, having media exposure, drugs given for intestinal parasites, and having child’s post-natal checkup were variables significantly associated with iron-rich food consumption. The region-based intervention will improve the consumption of iron-rich food. In addition, health policies and programs should target educating mothers/caregivers, encouraging parents to live together, improving their wealth status, working on mass media access by the women, and encouraging mothers post-natal checkups to improve iron-rich food consumption.

## Abbreviations

AOR: Adjusted Odds Ratio
RDHS: Rwanda Demographic Health Survey
ICC: Intra-class Correlation Coefficient
MOR: Median Odds Ratio
PCV: Proportional Change in Variance
WHO: World Health Organization
